# Editorial

**Published:** 1915-07

**Authors:** 


### EDITORIAL

The Atlanta Journal-Record of Medicine has its Business Office at 23 West Alabama Street where all business communications should be sent.

Remittances are payable to The Journal-Record of Medicine.

Concerning Original Communications and Editorial matters, address Richard R. Daly, M. D., 708 Empire Life Building, Atlanta, Ga.

Reprints of Original Articles will be furnished at cost price. Requests for them had best be made on the Manuscript.

Upon request, we will present, postpaid, to each contributor of an original article, twenty copies of The Journal-Record of Medicine containing such article.

### AGAIN TUBERCULOSIS.

In the symposium upon tuberculosis we publish to-day, the latest word has been said as to its etiology, diagnosis and treatment. The subject has been presented by men who are constantly handling the disease and who are striving to recognize every problem and to meet each successfully. No resource has been omitted and no effort spared to bring the care of these patients up to the limit set by scientific investigation when the patients have been under control. But the great number of sufferers are not under control and they do quite as they please in the matter of treatment and in disseminating their disease.

The old ideas of heredity are scarcely mentioned and the one time inevitable treatment with codliver oil was not spoken of at all. The attempt was made to get down to facts and to utilize them to the best advantage.

But there still remained what will be the incubus of the treatment of this disease and that is the freedom under the law of a patient to do as he pleases with himself and his infection.

Thus far the concerted efforts of private physicians and nurses and public institutions with their trained staffs have hardly kept pace with the spread of the disease. New cases are appearing each day in districts where great care is taken to help the people and the race is discouraging. It certainly will be to the strong in the end and tuberculosis will either be eradicated or do infinitely more damage according as to the strength of one side or the other of the contest.

If the disease is to be increased by permitting its sufferers to live as they please or as they are able in unsanitary surroundings and most deplorable housing conditions; if the patients are to be allowed to work in shops and stores and even homes where they are intimately associated with other people susceptible to the infection, then we shall always be trying to catch up and tuberculosis will be farther and farther in the lead and gather more and more of the prizes. If on the other hand, we take control in the beginning and by properly enacted laws isolate and quarantine every case of tuberculosis as we find it and search for it; if we keep it under strict control until we are certain it is no longer dangerous, then and not until then will science as she is now equipped be able to win the race; then will we be able to give correctly and fully the treatment each patient needs and at the same time stop the flood of new cases at its source.

We have ingrained the idea that a man's freedom must not be curtailed and that it would be a tremendous task to care for all the tuberculosis cases in a community. A tuberculosis patient now has freedom to do one thing more than any other and that is to spread his disease and to increase the suffering in the world. We do not hesitate in the least to deprive him of similar freedom when he has small pox or leprosy or yellow fever, why linger over protecting ourselves from vastly greater dangers? As to the task of caring for so many people, we have it anyhow because every tubercular case is somewhat of a burden upon the community, only we do not charge up the expense and do not see it. We might far better take care of him and them in ways calculated to assist them and at the same time let us know accurately what we are doing than to go on with the present death dealing method that has not even the merit of being business like.

Quarantine in public sanitoria every case of tuberculosis; give them every advantage science has to offer in the way of treatment; stop the spread of the disease to new patients and the race will be won. It is worth while.

